# Quality of the digital gp visits and characteristics of the users: retrospective observational study

**DOI:** 10.1080/02813432.2024.2380921

**Published:** 2024-07-21

**Authors:** Sanna Lakoma, Henna Pasanen, Kaisla Lahdensuo, Jaakko Pehkonen, Jutta Viinikainen, Paulus Torkki

**Affiliations:** a Department of Public Health, University of Helsinki, Helsinki, Finland; b Department of Economics, Jyväskylä University School of Business and Economics (JSBE), Finland; c Mehiläinen, Helsinki, Finland

**Keywords:** Primary health care, general practice, digital healthcare services

## Abstract

**Objectives:**

This study compares the demographics, diagnoses, re-admission rates, sick leaves, and prescribed medications of patients accessing digital general practitioner (GP) visits with those of patients opting for traditional face-to-face appointments in a primary health care setting.

**Design:**

The study adopted a retrospective analysis of patient record data collected in 2019, comparing visits to a digital primary health center with traditional health center visits.

**Setting:**

Primary health care.

**Participants:**

The data encompassed patients who utilized the digital clinic and those who visited public health centers for primary health care services.

**Main Outcome Measures:**

The study assessed demographics, health diagnoses, prescribed medications, sick leave recommendations, re-admission rates, and differences in costs between digital clinic and face-to-face visits. Secondary outcomes included a comparative analysis of medication categories, resolution rates for health problems, and potential impacts on health care utilization.

**Results:**

Digital clinic users were typically younger, more educated, and predominantly female compared with health centre users. Digital visits were well-suited for uncomplicated infections, while health centre appointments were associated with a higher prevalence of chronic conditions. Medication patterns differed between the two modalities, with digital clinic users receiving generic over-the-counter drugs and antibiotics, whereas health centre visits commonly involved cardiac and antihypertensive medications. Sick leave recommendations were slightly higher in the digital clinic, but the difference was not significant. Approximately 70% of health problems addressed in the digital clinic were successfully resolved, and the cost of digital visits was about 50,3% of face-to-face appointments.

**Conclusion:**

Digital health care services offer a cost-efficient alternative for specific health problems, appealing to younger, educated individuals, when compared to the users of public health center, and may enable improvement of cost-effectiveness combined with acceptable demand management and patient segmentation practices. The results highlight the potential benefits of digital clinics, particularly for uncomplicated cases, while also emphasizing the importance of suitable referral mechanisms for in-person consultations.

## Introduction

In recent years, the advancement of digital technologies has revolutionized health care delivery, providing new avenues for patients to access primary care services [1]. Digital health care services (DHS) have emerged as a convenient and efficient alternative to traditional face-to-face appointments, offering patients remote access to health care without the need to visit a health care facility [2]. As utilization of DHS continues to expand, it becomes crucial to understand the implications of this shift for patient demographics and diagnostic capabilities. It is important to elucidate the benefits and limitations of this evolving model of primary health care by investigating differences between users of regular general practitioners (GPs) and users of digital services.

The COVID-19 pandemic accelerated the adoption of DHS in both specialized and primary care [3]. However, the rapid implementation of digital services during the pandemic relied upon relatively scant scientific evidence regarding the benefits of these services or the perspectives of the patients and professionals utilizing them [4]. As health care systems have continued to offer digital services as an alternative or supplementary service to face-to-face consultations with primary care doctors after the pandemic [5], understanding the characteristics of patient groups becomes essential for health care providers and policymakers to tailor services effectively.

While DHS have been shown to be as effective as face-to-face consultations in primary care, there may be anomalies in quality factors, such as continuity of care, compared with traditional GP practices [6]. Additionally, studies have indicated that more antibiotic treatments are initiated for DHS users than for patients visiting a doctor’s office [7,8]. Nevertheless, DHS have demonstrated benefits in coordinating the care of chronically ill patients by improving self-care and follow-up for chronic diseases [9], and enabling active patient participation in their own care [2]. These services provide remote access to various treatments and assessments, addressing health care problems when face-to-face access is limited or costs are high [10].

Research on DHS has examined patient satisfaction, health effectiveness, service use, and cost at a global level [10]. Some studies suggest that DHS reduce the use of face-to-face health services, thereby improving access [11], while others indicate that DHS increase resource utilization and act as a duplication of services alongside physical services [12]. However, many studies have shown high patient satisfaction with DHS, with some patients even preferring DHS over face-to-face appointments [13,14]. Furthermore, evidence suggests that DHS can be cost-effective [15].

Such factors as age, sex, socioeconomic status, geographical location, and access to technology can significantly influence patient preferences. Previous research has highlighted variations in the user profiles of DHS, with younger, female, and higher educated individuals being more commonly associated with DHS utilization [16,17]. In contrast, older individuals and those with lower socioeconomic status often face barriers to accessing and utilizing DHS due to limited electronic health literacy, associated costs, and restricted internet access [18]. Consequently, concerns have been raised about the potential exclusion of certain patient groups from accessing or effectively utilizing DHS [19]. In addition, despite the implementation of DHS in primary and specialized health care settings, the heterogeneous group of patients in primary care with different health problems faces challenges when using these services [16,17]. Patients with chronic diseases significantly benefit from using digital services, such as patient portals [10], but long-term patients form a significant minority in primary health care relative to occasional users [20].

Digital health services have advantages such as accessibility regardless of patients’ geographical location and lower costs than face-to-face visits. Due to relatively easy access for those with the necessary digital skills, it may also reduce the underuse of health services. However, easy access may also lead to overuse of health care services, diverting resources from those who need them more. Determining which types of health care users can be reached by DHS and the health problems typically addressed remotely is the first step to understanding the pros and cons of DHS. To our knowledge, this is the first study comparing digital and face-to-face visits to GPs in primary health care in Finland.

### Objectives

The objective of this study is to compare a digital primary health centre (referred to as the "digital clinic") with a traditional primary health centre. Specifically, we compare the users of the digital clinic and their health problems with the users of traditional face-to-face visits at primary health centres. The main goal is to identify the patients and their health problems for which the digital clinic is best suited as a solution. Given that health-seeking behavior is influenced by factors such as gender and socioeconomic status, we aim to characterize the population utilizing the digital clinic. This information will assist in the design of digital services, ensuring that health disparities between different groups are not further exacerbated.

To achieve this, the study seeks to answer the following research questions:

1. Which patient groups choose digital primary care service and which patient groups choose face-to-face appointments? How do they differ in terms of their demographics?
2. What kinds of health problems/diagnoses could be solved digitally and what kinds require face-to-face visits?
3. Are there any differences in the medications prescribed or the amount of sick leave granted between the digital clinic and face-to-face visits?

This study primarily focuses on patients without chronic illnesses, and this patient segment will be referred to as “occasional patients” from here onwards. We aim to shed light on the characteristics and needs of this patient group in the context of DHS.

## Material and methods

### Background of the Finnish health system

The health care system in Finland is characterized by its comprehensive and equitable approach, providing universal access to high-quality medical services for all residents. The system is predominantly publicly funded, with the majority of health care services offered through public providers [21]. In Finland, there exists a notable degree of cost sharing, whereby approximately 20% of health expenditure is sourced from out-of-pocket payments [21]. Prior to 2023, the responsibility for organizing and delivering health care services rested with local municipalities. Commencing on 1 January 2023, a significant shift occurred, whereby the responsibility for orchestrating health care, social welfare, and rescue services were transferred from municipalities and joint municipal authorities to well-being service counties [22]. Primary care serves as the foundation of the health care system, and individuals are typically assigned to a specific primary care unit where they receive a wide range of services, including GP visits, preventive care, and health promotion. Specialist care is provided through referral from primary care physicians, and hospital services are available for more complex medical conditions. While public health care is the cornerstone of national health care, private health care providers also contribute to the system, offering additional options for those who seek expedited or specialized care [21].

### Case setting

This study was conducted in Finland and specifically examines the digital clinic, a digital public service within the primary care setting. Finland faces challenges regarding a shortage of health care professionals, particularly in the northern regions. However, limited access to primary health care and long waiting times are not exclusive to the northern areas [21]. Furthermore, being a sparsely populated country, with the majority of the population concentrated in the southern regions, the distances to health care centres can be considerable in certain parts of the country. Besides accessibility concerns, there are issues with maintaining continuity of treatment. To address these challenges, innovative care models have been developed, with DHS being one such solution.

The study population comprised the Länsi-Pohja area, situated in the northern region of Finland, with a total population of approximately 62,000 residents [23]. The digital clinic, which was operated by the private provider Mehiläinen, was outsourced to the municipalities of Tornio, Kemi, and Keminmaa in the Länsi-Pohja area, thus functioning as a public health care service. In the Länsi-Pohja area, patients have had the option of choosing between utilising the digital clinic or visiting the health centre for their health care needs since 1 April 2019. Users of the digital clinic had the convenience of accessing a non-scheduled appointment with a GP *via* a mobile application or internet browser chat, enabling almost round-the-clock contact. The GP provided care and addressed the patient’s concerns through chat or telephone communication. Physicians working at the digital clinic received training on the operational principles of the digital clinic, focusing on quality and safety. In cases where the issue could not be resolved remotely, the GP recommended an in-person scheduled visit to the health centre for further treatment. On the other hand, patients who opted to visit the health centre could make contact by telephone or by physically visiting the centre. In both scenarios, patients had the flexibility to reach out to their respective health care facilities for any health problem, and the services provided by the digital clinic were not limited to specific diagnoses or conditions.

### Data

Patient record data were collected for the period 1st April to 31 December 2019. During this time frame, we captured and analyzed all visits made to the digital clinic as well as face-to-face visits at the primary health care centre. Data obtained from patient records encompassed details of patients’ visits to either health centres or digital clinics, along with the corresponding diagnoses (ICD-10). Furthermore, the digital clinic data provided information on whether the patient’s issue could be resolved within the digital clinic or if they required referral to a health centre. The most prevalent visit diagnoses of patients were obtained from patient records. Since not all visits had an assigned diagnosis, the percentages of diagnoses were calculated by comparing them to visits for which a visit diagnosis was documented.

Patient record data were linked to Statistics Finland’s register information on demographic characteristics, including age, sex, occupation, and socioeconomic status, using unique personal identification numbers. Information on sick leaves was obtained from the Social Insurance Institution of Finland (Kela) registers, which include all sick absence periods lasting longer than 10 days. Data on sick leaves of shorter duration are not provided. If a GP certifies incapacity to work, the employer is obliged to notify Kela of the sick absence, and the employee is entitled to a daily sickness allowance paid by Kela after the initial 10-day period. Furthermore, information on medication purchases was collected from Kela registers, comprising all dispensed medicines reimbursed under the National Health Insurance scheme. The social insurance scheme administered by Kela is universal, covering all Finnish citizens. Data from Kela’s registers were linked to patient records and Statistics Finland’s data using unique identifiers.

The cost of a physical visit was defined as an average visit cost based on cost accounting system of the service provider. The cost of digital visit was based on unit price paid to the company by the service provider. The costs of digital clinic care were about 25% of the price of a health centre visit. Based on this, we calculated the cost differences between the digital clinic and health centres, with the cost of the digital clinic set at 25 units and the health centre at 100 units. Precise costs for this study were not available, and only the cost ratio was considered. Re-admission was defined as a subsequent visit of the same patient occurring within 1–14 days of the first visit.

All patients were included when examining differences in demographics and re-admissions between those utilizing the digital clinic and the health centre. Likewise, in evaluating the costs incurred from visits, all patients were considered. The focus on occasional patients was maintained, and patients with a diagnosed chronic conditions were excluded from the analysis when investigating distinctions in specific diagnoses, medications, and prescribed sick leaves. Chronic conditions, according to the Finnish Institute of Health and Welfare [24], include the following: cardiovascular diseases (I00–I99), diabetes (E10–E14), asthma and allergies (J45), chronic respiratory diseases (J40–J47), neoplasms (C00–D48), musculoskeletal diseases (M00-M99), and mental and behavioural disorders (F00–F99). Also, Z-diagnoses indicating mere interaction with the health care system (such as “other specified counselling” (Z71.8) or “persons encountering health services in other specified circumstances” (Z76.8)) were excluded from the analysis.

### Statistical analysis

We compared the characteristics, diagnoses, prescribed medications, and sick leaves of patients who had a digital clinic visit with those of patients having a face-to-face visit using means and percentages and either the Chi-square test (for categorical variables) or the Mann-Whitney U-test (for continuous variables) to assess statistical significance.

## Results

In total, 6997 visits were made to the digital clinic and 16,347 visits to health centres in the period 1 April to 31 December 2019. Table 1 presents the demographic characteristics of all patients accessing digital clinics compared with patients utilizing traditional health centres. Digital clinic users were predominantly female (*p* < 0.001) and were on average younger (*p* < 0.001) and had higher educational attainment than health centre users. In terms of health status, digital health care users generally demonstrated less chronic conditions than health centre users, as 44.52% (DHS 19.09%) of the latter reported having long-term illnesses (*p* < 0.001). A significant portion of health centre users was identified as pensioners. Re-admission rate for the digital clinic was 11.06% and for the health centre 8.85% (*p* < 0.001).

**Table 1.** Demographics and re-admission rate of the patients (1.4.–31 Dec 2019).

	User of digital clinic		User of GPs’ appointments		Digital vs. GP
			
	All patients	Occasional patients	All patients	Occasional patients	*p*-value
Number of visits	6997	5625	16347	8399	
Number of patients	2938	2377	10352	5743	
Proportion of long-term ill, %	19.09	NA	44.52	NA	*p* < 0.001
Male, %	30.00	3117	41.78	41.0	*p* < 0.001
Female, %	70.05	68.83	58.22	59.0	*p* < 0.001
Mean age, years (male)	23.33	19.72	49.55	36.12	*p* < 0.001
Mean age, years	32.24	29.1	51.85	40.53	*p* < 0.001
(female)					
Re-admission, %	11.06	0	8.85	0	*p* < 0.001
Re-admissions	774	0	1447	0	
Occupation (%)	Nurses (7.73)	Nurses (6.91)	Shop sales assistants (1.57)	Shop sales assistants (2.28)	
	Shop sales assistants (5.80)	Shop sales assistants (6.66)	Social work assistants (1.24)	Social work assistants (1.71)	
	Social work assistants (4.26)	Social work assistants (4.30)	Nurses (0.80)	Nurses (1.13)	
	Household service workers (2.86)	Household service workers (3.29)	Household service workers (0.91)	Household service workers (1.22)	
	Early childhood educators (2.20)	Early childhood educators (2.11)	Office cleaners (0.53)	Office cleaners (0.61)	
Socioeconomic status (%)	Other lower-level employees with administrative and clerical occupations (21.31)	Other lower-level employees with administrative and clerical occupations (21.41)	Pensioners (47.1)	Pensioners (25.14)	
	Manufacturing workers (12.88)	Manufacturing workers (14.53)	Other lower-level employees with administrative and clerical occupations (8.13)	Other lower-level employees with administrative and clerical occupations (11.28)	
	Distribution and service workers (8.44)	Distribution and service workers (8.70)	Long-term unemployed (7.91)	Students (10.08)	
	Clerical and sales workers, independent work (8.20)	Students (8.53)	Students (6.79)	Long-term unemployed (10.15)	
	Students (7.48)	Clerical and sales workers, independent work (8.40)	Manufacturing workers (6.12)	Manufacturing workers (9.77)	

Table 2 shows the most common diagnoses and medications among occasional patients. Within the digital clinic setting, the most frequently encountered occasional cases comprised uncomplicated infections such as conjunctivitis, acute upper respiratory infections, and acute cystitis (Table 2). On the other hand, the predominant diagnoses within health centre practices included such conditions as lower back pain, upper respiratory tract infections, ear infections, hypertension, and abdominal pain. Occasional patients utilizing the digital clinic had more prescriptions for antibiotics and mild painkillers (ibuprofen, amoxicillin, pivmecillinam, paracetamol, and cephalexin), while patients using the health centre had a greater proportion of prescriptions for medications used in chronic conditions (bisoprolol, salbutamol, and amlodipin in addition to analgesics).

**Table 2.** The most common visit diagnosis, prescribed medicines and sick leave-% among the occasional patients.

	Users of Digital Clinic, %		Users of GPs’ Appointments, %		*p*-value
Diagnosis	12.27	Conjunctivitis	6.30	Low back pain	
	9.16	Acute upper respiratory infection	4.43	Acute upper respiratory infection	
	8.18	Acute cystitis	3.32	Acute otitis media	
	2.26	Other gastroenteritis and colitis of infectious origin	2.62	Essential hypertension	
	1.76	Dermatitis, unspecified	2.56	Unspecified abdominal pain	
Medicines	8.17	Ibuprofein	6.07	Paracetamol	
	7.19	Amoxicillin	5.04	Ibuprofein	
	6.16	Pivmecillinam	2.60	Salbutamol	
	5.04	Paracetamol	2.57	Bisoprolol	
	4.84	Cephalexin	2.41	Amlodipin	
Sick-leaves %	10.40		8.37		NS

Among the occasional patients who sought care, sick leave records were found for 10.40% of digital clinic users and 8.37% of health centre users, but the difference was not statistically significant. Table 3 shows the most common diagnoses for sick leaves exceeding 10 days. Among the occasional patients of the digital clinic, the predominant diagnoses associated with sick leave were moderate depressive episodes, lower back pain, lower leg fracture, and false labour. These diagnoses differ from the most commonly observed conditions in the digital clinic setting. Conversely, sick leave at the health centre primarily pertained to lower back diseases and mental health problems.

**Table 3.** Sick-leaves Exceeding 10 days: Diagnosis and median length among the occasional patients.

Users of Digital clinic		Users of GPs’ appointments	
Diagnosis, % of all given	Median length (days)	Diagnosis, % of all given	Median length (days)
Lower back pain (5.06)	11	Moderate depressive episode (0.88)	50
Moderate depressive episode (3.80)	28	Lower back pain (0.79)	23.5
Fracture of lower leg (2,99)	26	Acute stress reaction (0.33)	38
False labour (2.53)	32	Other intervertebral disc disorders (0.31)	80.5

In the digital clinic, 71.77% of the cases were resolved and 26.56% had to be forwarded to face-to-face visits. The prevalent diagnoses among patients for whom the issue remained unresolved were conjunctivitis (9.92%), acute cystitis (8.26%), acute upper respiratory infection (7.44%), gastroenteritis (4.13%), and non-venomous insect bites (3.31%).

Table 4 shows the costs associated with the two modalities. As mentioned earlier, the costs of digital clinic care were about 25% of the price of a health centre visit. Based on this, we calculated the cost differences between the digital clinic and health centres, with the cost of the digital clinic set at 25 units and the health centre at 100 units. When calculating the cost difference between the digital clinic and the health centre, the costs for re-admitted patients were calculated twice. For the digital clinic, we also considered the costs of patients who had to be referred to a health centre visit after using the digital clinic. The costs for these patients comprised both the digital clinic visit and the subsequent health centre visit. The cost for the first visit to the digital clinic was estimated at 54.76 whereas for face-to-face appointments it was calculated to be 108.85.

**Table 4.** Total cost of the digital clinic and face-to-face appointments among all patients.

	Number of visits	Re-admission rate	Rate of patients sent to face-to-face appointments	Costs per visit index	Total costs per first visit
Digital clinic	6997	11.06%	27%	25	54.76
Face-to-face appointments	16,347	8.85%		100	108.85

## Discussion

Our findings provide insights into patient demographics, diagnoses, prescribed medications, and sick leave recommendations in digital GP visits compared with face-to-face appointments. Relatively little research has been conducted on the potential benefits of DHS and whether DHS can serve as a substitute for traditional face-to-face consultations. The initial step in understanding these issues is to determine who the users of digital services are and the circumstances under which digital services are utilized. This study partially fills this research gap.

Based on our results, digital clinic users in a public healthcare setting are predominantly female and are younger and have higher educational attainment than their counterparts. In contrast, health centre users are more likely to be pensioners and individuals experiencing long-term unemployment. Furthermore, a considerable proportion of health centre users (about 40%) appear to have chronic illnesses. These findings are consistent with previous research results [16,17].

Our study found that approximately 70% of the health problems addressed at the digital clinic were successfully resolved, while the remaining cases necessitated referral for face-to-face consultations. This proportion is relatively high, considering that patients had the option to contact the digital clinic for any health concern. However, it should be noted that our study did not include data on the duration of appointments at the digital clinic, which would have provided insight into time spent on these consultations.

The readmission rate for the digital clinic (11.06%) was significantly higher compared to face-to-face consultations (8.85%). A potential explanation for the increased re-admission rate observed in the digital clinic setting can be attributed to two primary factors. First, patients utilizing digital clinics may require several consultations, regardless of whether their initial visit was digital or in-person. As a result, the necessity for follow-up visits may be independent of the care delivery method. Second, it is possible that certain patient cases are too complex to be adequately addressed in a single digital consultation, necessitating further follow-up appointments. If these cases could have been managed in a traditional health center with fewer visits, it might have been a more cost-effective approach. Additional research is necessary to investigate the differences in re-admission rates and to understand the underlying causes.

We also found that the diagnoses encountered during digital GP visits and face-to-face consultations tended to be different. Common diagnoses at digital GP visits included uncomplicated infections, such as conjunctivitis, acute upper respiratory infections, and acute cystitis, which often rely on symptom-based assessments, visual examination, and patient-reported information. These diagnoses can arguably be effectively addressed through remote consultations, but they can also reflect the typical needs of those who seek help from digital services, i.e. the needs of young, well-educated, and relatively healthy individuals. It is noteworthy that the diagnosis code associated with the visit reason does not fully indicate the nature of the visit. For example, a visit related to a cardiac condition could involve either diagnosing the condition or adjusting medication dosage. The latter scenario is likely more suitable for a digital clinic visit, whereas the former might be too complex to resolve remotely. Our data does not include information on the reason for the visit at this level, but further studies could delve into this question.

We observed differences in medication prescriptions. The predominant medication categories for patients at the digital clinic comprise generic over-the-counter drugs and antibiotics suitable for uncomplicated infections. In contrast, patients attending in-person appointments commonly receive cardiac and antihypertensive drugs, suggesting a higher likelihood of treatment for chronic conditions in these settings. These variances may indicate that certain prescriptions can be reasonably and safely issued through a digital clinic, while others necessitate a face-to-face visit. Another explanation for the disparities is that individuals seeking care at the digital clinic exhibited specific patterns of medical needs and typically comprised relatively healthy, well-educated, and younger individuals with non-severe health conditions.

The data reveal that a higher proportion of individuals who received treatment at the digital clinic (10.40%) were prescribed a 10-day exceeding sick leave than individuals who visited a health centre (8.37%). However, the difference between the groups was not statistically significant, and this difference may be partly attributed to a large proportion of health centre users being retirees. It is important to note that definitive conclusions regarding sick leave-prescribing practices for each treatment modality cannot be drawn from the available data alone since there may be also shorter (less than 10 days) sick leaves that were not included in our data. Also, potential disparities in sick leave-prescribing practices may be attributed to the differing demographic and health status profiles of those who sought care through the digital clinic versus face-to-face appointments at the health centre. Patients may have opted for the digital clinic when immediate care or assistance was required, while in-person consultations at the health centre may have involved a greater number of follow-up visits. Further research is necessary to identify the factors contributing to this difference and to develop a comprehensive understanding of the specific needs addressed by the digital clinic and the health centre.

The findings indicate that the cost of digital clinics is approximately half of the cost of face-to-face appointments. When calculating the costs of digital clinic and primary care visits, re-admissions were considered, along with the acknowledgement that not all digital clinic visits could be resolved digitally; consequently, some patients had to be referred to face-to-face consultations. It should be noted that for the purposes of this study the exact monetary values were not obtained, but only the ratio between the costs was considered. Notably, the cost of face-to-face appointments only accounts for the physician’s labour and does not encompass the associated infrastructure expenses such as room rentals. Conversely, the cost of the digital clinic incorporates both the physician’s labour and the infrastructure costs. Therefore, the cost differential in favour of the digital clinic would likely be even more substantial in reality.

It is also worth noting that the digital clinic operates differently from the traditional Finnish health centre model. In the digital clinic, patients have direct access to a physician, whereas in the health center a nurse serves as a gatekeeper. Only a portion of the patients are handled directly by a physician at the health centre. This distinction in the health care delivery model between the digital clinic and the health centre may contribute to differences in cost per visit.

The literature presents conflicting findings regarding the effects of digital services on health care utilization. Some studies suggest that digital services do not significantly impact overall service use [25], while others indicate an increase in service utilization [12]. If service utilization increases after the introduction of a digital service option, it can imply either that there has been an underuse of health services due to difficulties with accessing face-to-face meetings or that easy access to digital health care services fosters overuse of health services among those with the necessary digital skills. If the introduction of digital health services leads to an overuse of health care services in certain patient groups, it is likely to result in suboptimal allocation of health care resources. This study was unable to assess whether the availability of the digital clinic resulted in heightened demand, i.e. whether patients were more inclined to seek care from the digital clinic due to its existence and direct access to a doctor. It would have been valuable to investigate whether direct access to a physician influenced the level of demand, particularly considering previous research indicating that while the need for in-person appointments may decrease with digital services, the easy accessibility of digital services could potentially lead to an increase in demand, particularly for direct-to-consumer services [26].

Earlier studies have shown that continuity of care may be weaker for primary care patients using digital services [6]. In this study, it was not possible to examine the continuity of care in the digital clinic, although this is an important and interesting topic. Future research should investigate the continuity of care in patients using digital services, both occasional patients and the chronically ill.

The main strength of our study is the data, which included comprehensive information on health care visits linked to administrative data on demographics, 10-day exceeding sick leaves, and medication use. However, some limitations should be considered. First, the data collected for this study are from the pre-pandemic era. During the COVID-19 pandemic, digital services were widely adopted across various health care sectors [27], which may have facilitated the transition to digitally managing various symptoms. Patients who used digital services in 2019 may also have been early adopters, and demographic differences to patients who used digital services after the pandemic may have levelled off. Since the users of the digital clinic were younger, their health problems were also different from those of the elderly patients utilizing health centres. Also, when considering the generalizability of the results, it is important to note that the findings are based on a single geographical region in Finland.

Second, our data were drawn in 2019, and since then, experience in treating patients in a digital clinic has accumulated, and patients can arguably be treated more often remotely. It is also possible that patients have learned over the years which conditions are suitable for treatment at the digital clinic. Patients with complex conditions may not even contact the digital clinic.

Third, data pertaining to patients’ sick leave and medication history have been sourced from databases maintained by the Social Insurance Institution of Finland. These datasets lack direct linkage to specific visits made to health centres or the digital clinic. Consequently, it is possible that in some cases a prescription or sick leave was erroneously linked to either a digital or face-to-face appointment. The data were collected over an 8-month time frame, which may be considered a relatively short duration, thus representing a limitation of the study. Also, the lack of data on under 10 days sick leaves may bias the results.

In conclusion, our study provides insights into the demographics, diagnoses, prescribed medications, and sick leave recommendations associated with digital GP visits and face-to-face appointments in primary health care. Understanding the characteristics and outcomes associated with each mode of care is essential for health care providers, policymakers, and researchers seeking to optimize health care delivery. By leveraging the strengths of digital health care while recognizing its limitations, the health care system can evolve towards a patient-centered and evidence-based approach that maximizes the benefits of both. Considering inclusion, patient groups with limited information technology skills and those with low electronic health literacy, should be equipped with the necessary abilities to utilize available digital health care services more widely. Previous studies have shown that prioritizing improved technical solutions, user-friendliness, and alignment with users are important for creating positive user experiences [28]. The confidence of patient who feel uncertain about digital health services can be improved if the digital services are easily usable, leading to higher usage rates [29]. While limited health literacy may pose challenges in digital health services it is probable that similar challenges exist during face-to-face meetings as well.

## Conclusions

A digital clinic represents a potentially cost-efficient approach for addressing specific medical conditions, particularly uncomplicated infections, and it may enable improvement of cost-effectiveness when combined with acceptable demand management and patient segmentation practices. Notably, in the public healthcare setting, the digital clinic model appears to align well with the preferences of younger individuals accustomed to using digital services. Attention should be paid to potential disparities in people’s readiness to utilize digital services, as the introduction of digital health services may impact differences in healthcare utilization and, therefore, long-term health outcomes.

## Data Availability

Data is available through a formal data request from the Finnish Institute for Health and Welfare.
